# Activation of the Extracytoplasmic Function σ Factor σ^V^ in Clostridioides difficile Requires Regulated Intramembrane Proteolysis of the Anti-σ Factor RsiV

**DOI:** 10.1128/msphere.00092-22

**Published:** 2022-03-23

**Authors:** Anthony G. Pannullo, Craig D. Ellermeier

**Affiliations:** a Department of Microbiology and Immunology, Carver College of Medicine, University of Iowa, Iowa City, IA, USA; b Graduate Program in Genetics, University of Iowa, Iowa City, IA, USA; University of Michigan-Ann Arbor

**Keywords:** σ factors, cell envelope, stress response, signal transduction, gene expression, sigma factors

## Abstract

Clostridioides (Clostridium) difficile is one of the leading causes of nosocomial diarrhea. Lysozyme is a common host defense against many pathogenic bacteria. C. difficile exhibits high levels of lysozyme resistance, which is due in part to the extracytoplasmic functioning (ECF) σ factor, σ^V^. It has been previously demonstrated that genes regulated by σ^V^ are responsible for peptidoglycan modifications that provide C. difficile with high lysozyme resistance. σ^V^ is not unique to C. difficile however, and its role in lysozyme resistance and its mechanism of activation has been well characterized in Bacillus subtilis where the anti-σ, RsiV, sequesters σ^V^ until lysozyme directly binds to RsiV, activating σ^V^. However, it remains unclear if the mechanism of σ^V^ activation is similar in C. difficile. Here, we investigated how activation of σ^V^ is controlled in C. difficile by lysozyme. We found that C. difficile RsiV was degraded in the presence of lysozyme. We also found that disruption of a predicted signal peptidase cleavage site blocked RsiV degradation and σ^V^ activation, indicating that the site-1 protease is likely a signal peptidase. We also identified a conserved site-2 protease, RasP, that was required for site-2 cleavage of RsiV and σ^V^ activation in response to lysozyme. Combined with previous work showing RsiV directly binds lysozyme, these data suggested that RsiV directly binds lysozyme in C. difficile, which leads to RsiV destruction via cleavage at site-1 by signal peptidase and then at site-2 by RasP, ultimately resulting in σ^V^ activation and increased resistance to lysozyme.

**IMPORTANCE** Clostridioides difficile is a major cause of hospital-acquired diarrhea and represents an urgent concern due to the prevalence of antibiotic resistance and the rate of recurrent infections. We previously showed that σ^V^ and the regulon under its control were involved in lysozyme resistance. We have also shown in B. subtilis that the anti-σ RsiV acts as a direct sensor for lysozyme. which results in the destruction of RsiV and activation of σ^V^. Here, we described the proteases required for degradation of RsiV in C. difficile in response to lysozyme. Our data indicated that the mechanism is highly conserved between B. subtilis and C. difficile.

## INTRODUCTION

Clostridioides difficile is an anaerobic Gram-positive opportunistic pathogen that is the most common cause of hospital-associated diarrhea. C. difficile causes approximately 220,000 infections and 12,800 deaths in the US each year, with an estimated annual cost of $1 billion in medical expenses (1). C. difficile infections (CDI) typically affect people who have taken antibiotics which disrupt the normal microflora of the gastrointestinal tract resulting in dysbiosis. Dysbiosis is important as it is thought that the healthy gut microflora prevents C. difficile colonization or at least prevents the progression of the disease (2, 3). C. difficile spores are highly resistant to antibiotic therapy and, upon germination, C. difficile colonizes the colon before a healthy microbiota can be reestablished (4, 5). The pathology of CDI is largely attributed to Toxin A and Toxin B, which glucosylate Rho GTPases. The toxins destroy the intestinal epithelium, inflammation, diarrhea, and, in some cases, lead to toxic megacolon (6, 7).

To survive, bacteria need to be able to adapt to a wide variety of environments each with unique stressors. Bacteria often express the genes required for stress responses only when required. Thus, many bacteria have developed specialized signaling systems to detect and respond to environmental stressors. These signaling systems can be quite varied and include large groups such as one-component systems, two-component systems, alternative sigma (σ) factors, and extracytoplasmic functioning (ECF) σ factors (8–12). ECF σ factors are a group of σ factors that are often involved in responding to external signals (8, 9, 13). ECF σ factor activity is often inhibited by a cognate anti-σ, which sequesters the ECF σ from RNA polymerase (RNAP) until the appropriate signal is detected. Once the signal is detected, the ECF σ is released from the anti-σ, via a variety of different mechanisms, including conformational change of the anti-σ which leads to release of the σ factor, a partner switching mechanism in which the anti-σ binds to a different substrate, which frees the ECF σ factor, and regulated intramembrane proteolysis (RIP) where the anti-σ is degraded by a series of proteases (14–17).

C. difficile encodes the ECF σ factor σ^V^, which is responsible for detection and response to the innate host-factor lysozyme (18–20). σ^V^ controls expression of the peptidoglycan (PG) deacetylase PdaV, which, in conjunction with the peptidoglycan (PG) deacetylase PgdA, is responsible for conferring resistance to lysozyme in C. difficile (20, 21). This resistance is due to the deacetylation of the *N*-acetylglucosamine (NAG) residues of the PG. Under normal circumstances, lysozyme recognizes the repeating NAG and *N-*acetylmuramic acid (NAM) backbone of the PG and cleaves the β1-4 linkage between NAG and NAM resulting in cell lysis. However, when NAG residues become deacetylated lysozyme has a lower affinity for the PG backbone, which prevents lysozyme-mediated lysis (22). In C. difficile, when σ^V^ is fully activated approximately 90 to 95% of the NAG residues exist in a deacetylated state, providing resistance up to 16 mg/mL of lysozyme (18, 20, 21). In addition to *pdaV*, another gene found in the *csfV* operon, *lbpA*, has been shown to influence lysozyme resistance (20). LbpA is a RsiV ortholog and has been shown to bind lysozyme, but it lacks the σ^V^ binding domain and, thus, does not play a role in σ^V^ activation or regulation (20). However, it does act as a membrane-bound lysozyme binding protein that can confer resistance to low levels of lysozyme (20). It has also been shown that σ^V^ regulates the expression of lysozyme resistance genes outside the *csfV* operon, including the *dltABCD* operon. The *dlt* operon is partially regulated by σ^V^, and, thus, responds to lysozyme. The *dlt* operon mediates lysozyme resistance through d-alanylation of lipid teichoic acids that protrude from the PG (23). This d-alanylation is predicted to drive the charge of the cell envelope to become less negative and reduce the affinity that lysozyme has for the PG (23). It has been shown that CRIPSRi knockdown of the *dlt* operon in C. difficile results in a ∼16-fold decrease in lysozyme MIC (20).

σ^V^ is present in many Gram-positive bacteria, including the model organism Bacillus subtilis, where extensive work has been done to understand the mechanism of σ^V^ activation (24–29). In B. subtilis the anti-sigma factor of σ^V^, RsiV, binds directly to lysozyme. The C. difficile and Enterococcus faecalis RsiV orthologs also directly bind lysozyme (29). Lysozyme binding is predicted to lead to conformational change within RsiV that initiates RIP of RsiV (25, 26). RIP generally consists of two-step proteolysis that utilizes a site-1 protease (S1P) and a site-2 protease (S2P) (13, 15, 30). In B. subtilis it was found that the S1P for RsiV are type 1 signal peptidases, specifically the major type 1 signal peptidases in B. subtilis SipS and SipT (27). Site-1 cleavage removes the extracellular portion of RsiV. However, the membrane-bound portion remains and continues to serve as a membrane anchor for σ^V^, preventing activation. A second cleavage event is required to release RsiV from the membrane. This is carried out by the intramembrane protease or S2P, which in B. subtilis is RasP (28). RasP is a conserved metalloprotease that exists in many bacteria and eukaryotes (30, 31). In addition to cleaving anti-σ factors, S2Ps also cleave remnant signal peptides to clear them from the membrane (32). After site-2 cleavage the remaining portion of RsiV complexed with σ^V^ is released into the cytosol, the vestiges of RsiV bound to σ^V^ are degraded likely via cytosolic proteases and σ^V^ is free to regulate its operon. This includes *oatA*, which is found in B. subtilis but not in C. difficile and encodes a peptidoglycan *O-*acetyltransferase, which increases lysozyme resistance by acetylating MurNac when expressed (24, 33, 34). While we know the mechanism of σ^V^ activation in B. subtilis, we do not know if this mechanism is conserved in C. difficile. In this study, we investigated the degradation of RsiV in response to lysozyme. We identified a C. difficile site-2 protease homolog, RasP, and showed that it was required for σ^V^ activation, lysozyme resistance, and RsiV degradation. We also demonstrated that C. difficile RsiV has a signal peptidase cleavage site and mutations in this site block RsiV degradation.

## RESULTS

### C. difficile RsiV is degraded in the presence of lysozyme.

**FIG 1:**
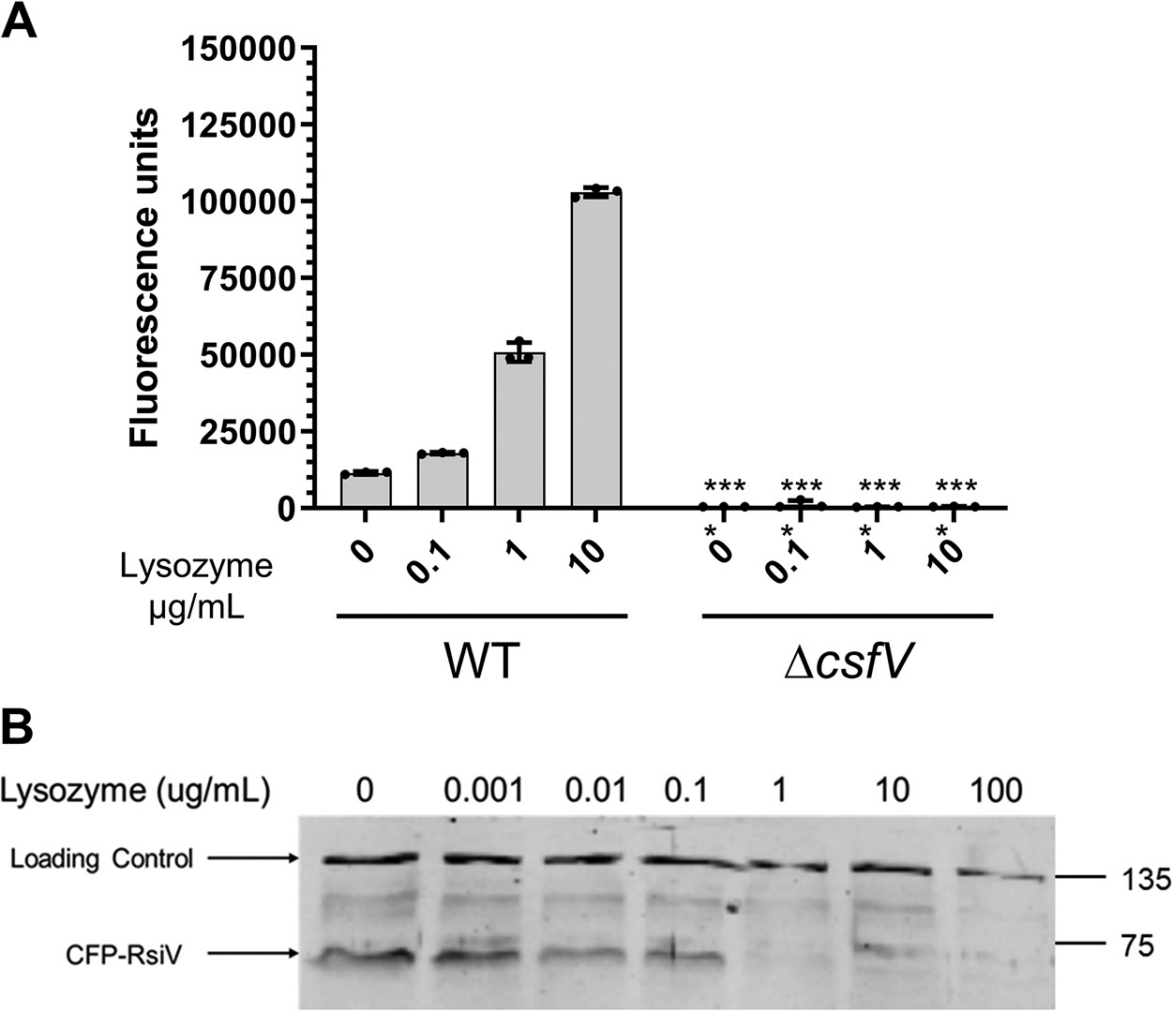
σ^V^ responded to lysozyme and RsiV was degraded in the presence of lysozyme in C. difficile. (A) The P*_pdaV_*-RFP reporter was used to measure activation of the σ^V^ in response to increasing concentrations of lysozyme. WT and Δ*csfV* that contained pRAN738 (P*_pdaV_*-RFP) were grown to an OD_600_ of 0.3. Cells were then induced with lysozyme for 2 h and removed from the anaerobic chamber. Samples were fixed and fluorescence was allowed to develop overnight. Data were analyzed by two-way analysis of variance with Sidak’s multiple-comparison test. ****, *P* < 0.0001; ***, *P* < 0.001; **, *P* < 0.001 (comparisons were made to the wild-type strain with corresponding lysozyme concentration unless otherwise noted by a black bar). (B) Western blot showing degradation of CFP-RsiV is lysozyme dependent and occurs in a dose-dependent manner. Numbers on the right indicated masses in kilodaltons.

We previously demonstrated that C. difficile *csfV*, which encodes σ^V^, is activated by lysozyme and is required for lysozyme resistance (18, 20). In B. subtilis and E. faecalis activation of σ^V^ requires the degradation of the anti-sigma factor, RsiV (28, 29, 35, 36). To demonstrate lysozyme-mediated activation of σ^V^ in C. difficile, we introduced a P*_pdaV_*-RFP reporter plasmid in both wild-type (WT) and Δ*csfV* strains. We incubated cells with increasing concentrations of lysozyme and measured the fluorescence. As previously reported, we found P*_pdaV_* was induced by lysozyme in the WT and expression increased in response to increasing lysozyme concentrations (Fig. 1A) (20). In a Δ*csfV* mutant, however, expression of the P*_pdaV_* reporter was significantly lower than WT and was no longer induced in response to lysozyme (Fig. 1A) (20). To determine if σ^V^ activation correlated with lysozyme-induced degradation of RsiV in C. difficile, we constructed a P*_xyl_*-CFP-RsiV fusion protein. This allowed us to detect the fate of the N-terminal portion of RsiV and uncouple its production from σ^V^ activity because CFP-RsiV fusion would be induced by σ^V^ if expressed from its native promoter. We then monitored CFP-RsiV levels in cells incubated with increasing concentrations of lysozyme for 15 min. We found that CFP-RsiV levels decreased as lysozyme levels increased (Fig. 1B). Given the short time of incubation with lysozyme, it suggested that RsiV was degraded and this could be detected around 0.1 to 1 μg/mL of lysozyme (Fig. 1B). We note that decreasing RsiV levels and increasing σ^V^ activation appeared at similar lysozyme concentrations, suggesting that degradation of RsiV controls σ^V^ activation and expression of P*_pdaV_*. This is well below the 8 to 16 mg/mL MIC of lysozyme, suggesting σ^V^ activation occurred at subinhibitory lysozyme concentrations (20).

### Degradation of RsiV was dependent upon the presence of a signal peptidase cleavage site.

RIP of an anti-σ factor is a common mechanism for activation of an ECF sigma (9, 13, 37). RIP consists of two major proteolytic events by site-1 and site-2 proteases (S1P, S2P), respectively. We previously identified the two major signal peptidases SipS and SipT as the proteases responsible for site-1 cleavage of RsiV in B. subtilis (27). In B. subtilis, site-1 cleavage of RsiV occurred at a signal peptide cleavage site located at the junction of the transmembrane and extracytoplasmic domains. The cleavage site is the typical ‘A-X-A’ motif present in most proteins cleaved by signal peptidases in B. subtilis (38). It should be noted that cleavage occurred directly after the recognized cleavage motif and not within the cleavage motif itself. We hypothesized that RsiV degradation in C. difficile functioned in a similar if not identical manner to B. subtilis.

**FIG 2:**
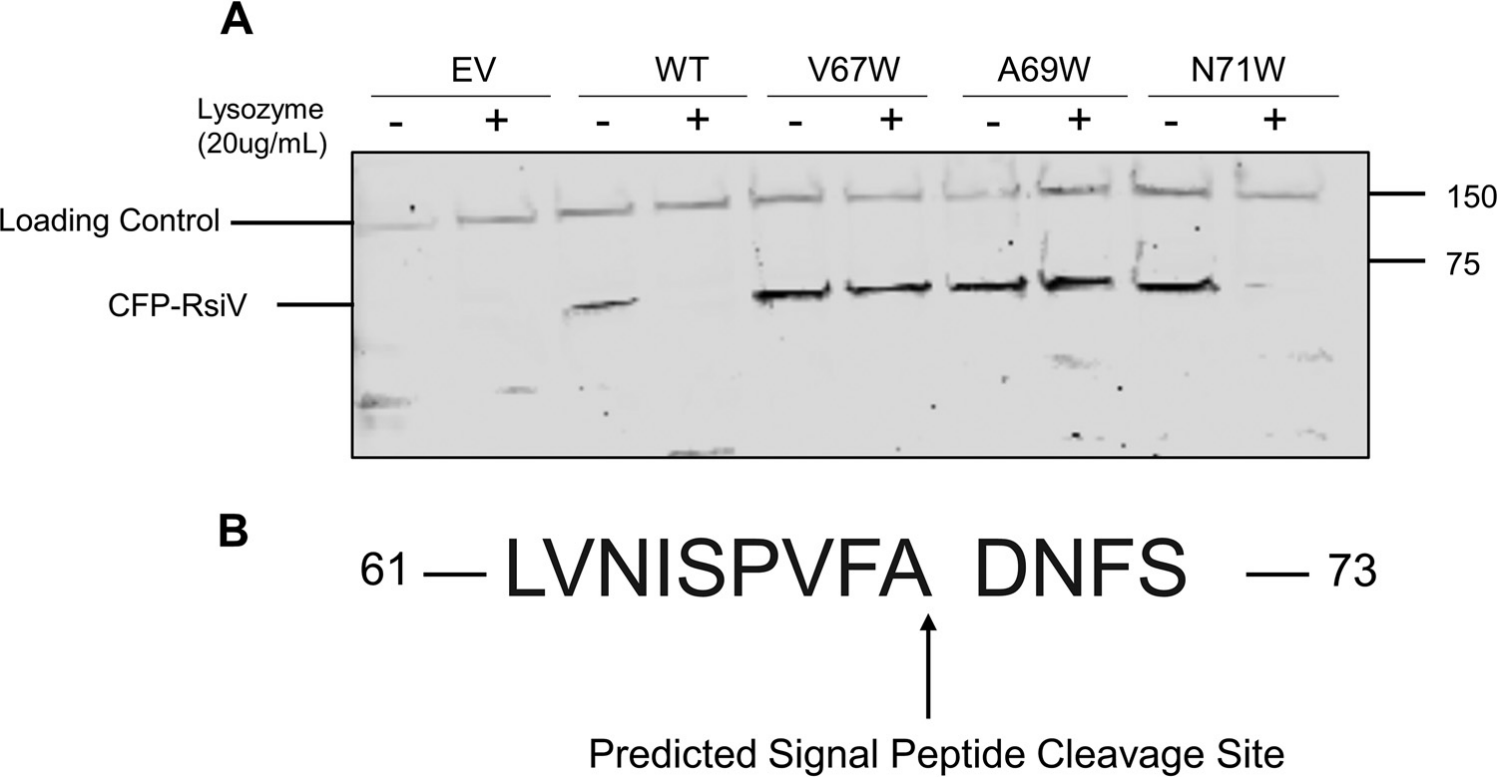
Degradation of RsiV is dependent upon the presence of signal peptidase cleavage site. (A) Western blot showing that lysozyme-dependent degradation of CFP-RsiV required the signal peptidase cleavage site VFA, which followed the ‘V-X-A’ motif recognized by some signal peptidases. WT cells containing different CFP-RsiV mutant plasmids, WT (pCE620), V67W (pCE627), A69W (pCE621), or N71W (pCE622) were grown in TY + 1% xylose to mid-log phase (OD_600_ ∼0.7) Numbers on the right indicate masses in kilodaltons. (B) Amino acid sequence of residues 61 to 73 in C. difficile (R20291) RsiV. The predicted signal peptide cleavage site between residues A69 and D70 is indicated by the arrow.

We analyzed the sequence of RsiV using SignalP (39). We also performed a sequence alignment of RsiV from B. subtilis and C. difficile using Clustal Omega (40), focusing on the relative location of the B. subtilis signal peptide cleavage site. The alignment of the cleavage sites suggests that C. difficile RsiV could be cleaved between residues N71 and F72 (Fig. S1A). This would make the signal peptidase motif “A-X-N’. In contrast, SignalP predicts the C. difficile RsiV cleavage site to be between residues A69 and D70 which would be a ‘V-X-A’ motif (Fig. 2B and Fig. S1B).

10.1128/msphere.00092-22.1 FIG S1 (A) Alignment of C. difficile RsiV and B. subtilis RsiV. The red box highlights the putative cleavage site. (B) SignalP-5.0 output for C. difficile RsiV showing ∼70% probability that the V-F-A sequence is a signal peptide cleavage site. Blue line is C. difficile RsiV and red line is B. subtilis RsiV. Download FIG S1, TIF file, 2.0 MB. Copyright © 2022 Pannullo and Ellermeier. 2022 Pannullo and Ellermeier. https://creativecommons.org/licenses/by/4.0/ This content is distributed under the terms of the Creative Commons Attribution 4.0 International license.

We sought to disrupt the putative signal peptide cleavage sites by introducing tryptophan residues at the putative −3, −1, and +1 positions to determine if changes to these residues blocked the degradation of RsiV. We constructed four variants of P_xyl_-CFP-RsiV, which included WT, V67W, A69W, and N71W. Substitutions that convert the residues to bulky tryptophans will prevent recognition of the cleavage site, and it was shown to successfully block cleavage when performed in B. subtilis (29). We introduced these constructs into C. difficile and found that the WT and N71W mutant versions of CFP-RsiV were degraded in the presence of 20 μg/mL lysozyme (Fig. 2A). In contrast, the V67W and A69W mutants blocked the degradation of CFP-RsiV in the presence of lysozyme (Fig. 2A). This suggested that the ‘V-X-A’ motif and not the ‘A-X-N’ motif was likely the site-1 cleavage site. This also suggested that the cleavage site was likely between A69 and D70. This was consistent with the SignalP prediction and suggested that signal peptidases were the S1P for RsiV in C. difficile as they are in B. subtilis.

### The site-2 protease RasP was required for complete degradation of RsiV and maximal activation of σ^V^.

As previously described, RIP mediated degradation of anti-σ factors requires two proteolytic events, the second of which is performed by the conserved, membrane-embedded S2P (31). In B. subtilis, RasP is required for RIP mediated degradation of anti-σ factors, including RsiW and RsiV (28, 41, 42). Because S2P are highly conserved, we used B. subtilis RasP to search for potential C. difficile S2P homologs using BLASTP (43). BLASTP revealed CDR20291_2036 (referred to as RasP) shared 38% amino acid identity to B. subtilis RasP (Fig. S2). Importantly, the metalloprotease active site motif, HEXXH, was highly conserved across different species as shown in Fig. S2, indicating that the two proteins likely function in a similar manner.

10.1128/msphere.00092-22.2 FIG S2 Alignment of B. Subtilis, C. difficile, E. faecalis, and E. coli RasP Homologs. The conserved active site motif HEXXH is highlighted by a red box. Download FIG S2, TIF file, 1.8 MB. Copyright © 2022 Pannullo and Ellermeier. 2022 Pannullo and Ellermeier. https://creativecommons.org/licenses/by/4.0/ This content is distributed under the terms of the Creative Commons Attribution 4.0 International license.

To determine if RasP was required for site-2 cleavage of RsiV in C. difficile, we constructed an in-frame deletion of *rasP* in the R20291 background using CRISPR editing (44). This deletion strain is referred to as Δ*rasP*. Using this deletion, we compared P*_pdaV_*-RFP reporter activation, lysozyme MICs, and CFP-RsiV degradation in the WT and Δ*csfV* strains to determine the role that RasP plays in σ^V^ signaling.

**FIG 3:**
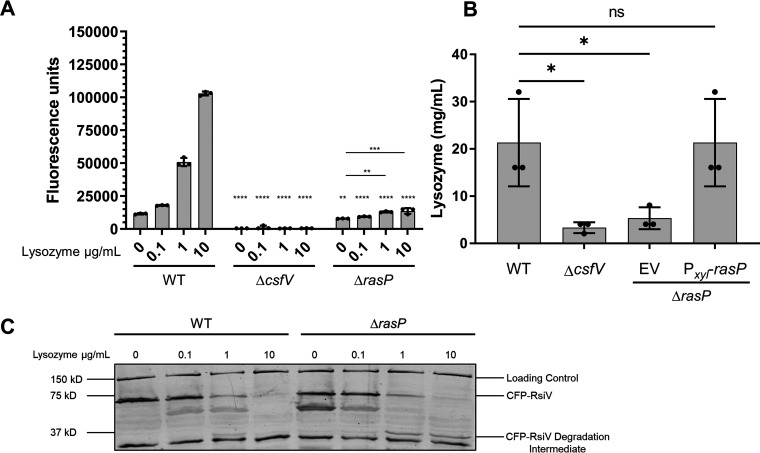
The site-2 protease RasP is required for degradation of RsiV and maximal activation of σ^V^. (A) Strains containing the P*_pdaV_*-RFP (pRAN738) reporter WT (AP160), Δ*csfV* (GMK211), and Δ*rasP* (AP153) were grown to an OD_600_ of 0.3. Cells were then induced with lysozyme for 2 h and removed from the anaerobic chamber, fixed, and allowed to fluoresce overnight Data were analyzed by two-way analysis of variance with Sidak’s multiple-comparison test. **, *P* < 0.01; ****, *P* < 0.0001 (compared to the wild-type strain with corresponding lysozyme concentration). (B) Lysozyme MIC with WT, Δ*csfV*, and Δ*rasP* strains. Complementation of *rasP* was done using P_xyl_-RasP (pCE676) in which 1% xylose was included in the TY growth medium. Data were analyzed by one-way analysis of variance with Sidak’s multiple-comparison test. NS, *P* > 0.05; *, *P* < 0.05. (C) Western blot showing that lysozyme dependent-degradation of CFP-RsiV required the site-2 protease RasP. The absence of RasP led to the formation of an intermediate cleavage product. WT (AP136) or Δ*rasP* (AP151) containing CFP-RsiV were grown in TY + 1% xylose to mid-log phase (OD_600_ ∼0.7). Numbers on the right indicate masses in kilodaltons.

We found that the basal level activity of the P*_pdaV_*-RFP reporter was reduced in the Δ*rasP* strain compared to the WT (Fig. 3A). However, P*_pdaV_*-RFP activity was higher in Δ*rasP* compared to Δ*csfV* (Fig. 3A). Expression of the P*_pdaV_*-RFP activity was not significantly induced in response to low concentrations of lysozyme in the Δ*rasP* mutant (Fig. 3A). However, at higher concentrations of lysozyme, the increase in reporter activity in the Δ*rasP* mutant became significantly different compared to the no lysozyme control for the strains (Fig. 3A). However, this increase in reporter fluorescence was lower than the increase in fluorescence that was observed in the WT, indicating that RasP was required for maximal activation of the *csfV* operon (Fig. 3A).

We found the Δ*rasP* mutant also displayed a lower lysozyme MIC (4 to 8 mg/mL) compared to WT (approximately 16 to 32 mg/mL) (Fig. 3B). The decrease in lysozyme resistance of the Δ*rasP* mutant could be complemented by ectopic expression of *rasP* in the Δ*rasP* strain in which the lysozyme resistance was restored to the WT levels (Fig. 3B).

To determine if RasP was required for site-2 cleavage, we asked if the *ΔrasP* mutant led to the accumulation of the site-1 cleavage product. We expressed CFP-RsiV in the wild-type and *ΔrasP* mutant and then split the cultures to be treated with a range of lysozyme concentrations for 15 min. We found that treatment with lysozyme led to a buildup of partially cleaved CFP-RsiV in the *ΔrasP* mutant, which can be observed by the band that corresponds to ∼30 kDa. This was consistent with the loss of site-2 protease activity (Fig. 3C). However, the loss of RasP did not appear to completely block site-2 cleavage of RsiV because the buildup of the intermediate product was not equivalent to the level of full-length RsiV in untreated cells. Taken together, these data suggested that RasP was required for optimal cleavage of RsiV at site-2 and, thus, σ^V^ activation in C. difficile. However, in the intermediate lysozyme resistance phenotype, the higher basal level of σ^V^ activity suggested another protease may be capable of cleaving RsiV at site-2.

## DISCUSSION

In this work, we demonstrated that, in C. difficile, RsiV degradation and subsequent σ^V^ activation in the presence of lysozyme functions in a similar fashion as previously described in B. subtilis (27–29). We showed that in C. difficile RsiV was degraded in the presence of lysozyme in a dose-dependent manner. We also showed that, in C. difficile, RsiV was degraded through a RIP-mediated process similar to that in B. subtilis (27–29). In B. subtilis, the S1P is signal peptidase, more specifically SipS and/or SipT, is a type-I signal peptidase (27). These peptidases were shown to cleave B. subtilis RsiV at a predicted signal peptide cleavage site, which follows a canonical ‘A-X-A’ motif commonly seen in B. subtilis secreted proteins (38). Here, we showed that C. difficile RsiV was likely cleaved at a signal peptide cleavage site, albeit of a slightly different motif than the one found in B. subtilis. Changing the residues in the signal peptide sequence to tryptophan residues completely prevented lysozyme-induced degradation of RsiV. The most likely explanation is that the signal peptidases are no longer able to recognize the cleavage site when tryptophan is present. It is interesting to note that the C. difficile signal peptide sequence is closer to the transmembrane domain than in B. subtilis however the functional consequence of that remains unclear.

C. difficile encodes three putative type-I signal peptidases. We have not established the signal peptidases in C. difficile that are responsible for site-1 cleavage of RsiV. Considering the conserved nature of the RsiV degradation and activation mechanisms between C. difficile and B. subtilis it seems reasonable to hypothesize that the signal peptidases in C. difficile could be redundant just as in B. subtilis. This is supported by Tn-seq data showing that none of the type-1 signal peptidases are essential as each of the putative signal peptidase encoding genes had multiple transposon insertions, suggesting there is functional redundancy as in B. subtilis (45, 46).

We identified a RasP homolog based on conservation. However, it is worth noting that alignment of C. difficile RasP to B. subtilis RasP (Fig. S2) revealed that, while there is a great deal of homology, there are also some distinct differences. B. subtilis RasP and E. faecalis Eep contains an 89 amino acid insertion in a cytoplasmic domain (Fig. S2). This insertion is absent in both C. difficile RasP and E. coli RseP. RseP in E. coli has two distinct PDZ domains on the extracellular face where RasP from C. difficile, B. subtilis, and E. faecalis has a single PDZ domain. Since RasP is a highly conserved protease, even across different domains of life, it seemed very likely that RasP would be conserved in the degradation of RsiV in C. difficile. Indeed, our Δ*rasP* mutant shows decreased σ^V^ activity in the presence of lysozyme compared to our WT. We were also able to detect the accumulation of the RsiV degradation intermediate in the absence of RasP suggesting it is required for site-2 cleavage. Interestingly, the Δ*rasP* mutant appears to exhibit intermediate phenotypes in both the P*_pdaV_*-RFP reporter and lysozyme MIC assays. In B. subtilis, the activity of σ^V^ in a *rasP* mutant is identical to the loss of σ^V^ itself (28). However, in C. difficile this was not the case. We saw that, compared to a *csfV* mutant, a *ΔrasP* mutant had higher basal levels of σ^V^ activity. In addition, we saw the induction of σ^V^ activity in the presence of lysozyme in the *ΔrasP* mutant, but the fold change was much lower than what occurs in the WT. We also observed that the RsiV intermediate product band does not appear to become more intense with higher concentrations of lysozyme, which would be the anticipated phenotype. We hypothesize that other proteases can cleave RsiV at site-2. However, these proteases are less efficient. This is supported by our data showing Δ*rasP* mutants display intermediate phenotypes in the expression of P*_pdaV_-rfp* and sensitivity to lysozyme as well as a buildup of the site-1 cleavage product of RsiV just not to levels of the full-length band. While there is a large number of similarities with σ^V^ activation in B. subtilis the presence of additional proteases that can cleave RsiV at site-2 is distinct. Additional work will be required to identify the other protease(s) in C. difficile that can function as a site-2 protease for RsiV in the absence of RasP.

## MATERIALS AND METHODS

### Bacterial strains, media, and growth conditions.

Bacterial strains are listed in Table 1. The C. difficile strains used in this study were derivatives of R20291. C. difficile strains were grown on tryptone-yeast (TY) medium supplemented as needed with thiamphenicol at 10 μg/mL (Thi_10_) kanamycin at 50 μg/mL and cefoxitin at 50 μg/mL. TY consisted of 3% tryptone, 2% yeast extract and 2% agar (for solid medium). C. difficile strains were maintained at 37°C in an anaerobic chamber (Coy Laboratory Products) in an atmosphere of 10% H_2_, 5% CO_2_, and 85% N_2_.

**TABLE 1:** Strains

Species and strain	Genotype and/or description	Source or reference^*^
**E. coli**		
OmniMAX-2 T1R	*F′ {proAB+ lacIq lacZΔM15 Tn10(TetR) Δ(ccdAB)} mcrA Δ(mrr-hsdRMS-mcrBC) φ80(lacZ)ΔM15 Δ(lacZYA-argF) U169 endA1 recA1 supE44 thi-1 gyrA96 relA1 tonA panD*	Invitogen
HB101/pRK24	*F− mcrB mrr hsdS20(rB− mB −) recA13 leuB6 ara- 14 proA2 lacY1 galK2 xyl-5 mtl-1 rpsL20*	(51)
MG1655	Wild type	
**B. subtilis**		
BS49	Tn916 donor strain, TetR	(52)
**C. difficile**		
R20291	Wild-type strain from UK outbreak (ribotype 027)	(53)
CDE2966	R20291 Δ*csfV*	(20)
AP150	R20291 Δ*rasP*	
GMK208	R20291/pRAN738 (P*_pdaV_*-*rfp*)	(20)
GMK211	R20291 Δ*csfV*/pRAN738 (P*_pdaV_*-*rfp*)	(20)
AP153	R20291 Δ*rasP*/pRAN738 (P*_pdaV_*-*rfp*)	
AP136	R20291/pCE620 (P*_xyl_*-CFP-RsiV)	
AP151	R20291 Δ*rasP/*pCE620 (P*_xyl_*-CFP-RsiV)	
AP441	R20291/pAP114 (P*_xyl_*-*rfp*)	
AP230	R20291 Δ*rasP*/pCE675 (P*_xyl_*-*rasP*)	
AP131	R20291/pCE627 (P*_xyl_*-CFP-RsiV^V67W^)	
AP126	R20291/pCE621 (P*_xyl_*-CFP-RsiV^A69W^)	
AP127	R20291/pCE622 (P*_xyl_*-CFP-RsiV^N71W^)	

* Unless noted strains were generated as part of this study.

E. coli strains were grown in LB medium at 37°C with chloramphenicol at 10 μg/mL and ampicillin at 100 μg/mL as needed. LB contained 1% tryptone, 0.5% yeast extract, 0.5% NaCl, and 1.5% agar (for solid medium).

### Plasmid and bacterial strain construction.

All plasmids are listed in Table 2 and Table S1. Plasmids were constructed using Gibson Assembly (New England Biolabs, Ipswich, MA). Regions of the plasmids constructed using PCR were verified by DNA sequencing. Oligonucleotide primers used in this work were synthesized by Integrated DNA Technologies (Coralville, IA) and are listed in Table S2. All plasmids were propagated using OmniMax-2 T1R as a cloning host. CRIPSR-Cas9 deletion plasmids were passaged through Escherichia coli strain MG1655 before transformation into B. subtilis strain BS49. CRISPR-Cas9 plasmids were built on the backbone of pJKO2 (44) with some modifications (20).

**TABLE 2:** Plasmids

Plasmid	Relevant features	Reference
pRPF185	E. coli*-*C. difficile shuttle vector with the tetracycline-inducible promoter; P*_tet_::gusA cat CD6ori RP4oriT-traJ pMB1 ori*	(54)
pRAN738	P*_pdaV_*::*mCherryOpt cat*	(20)
pAP114	P*_xyl_*::*mCherryOpt cat*	(47)
pRAN357	P*_tet_*::*cfp cat*	(48)
pCE641	P*_xyl_::cas9-opt ΔrasP* P*_gdh_::sgRNA-rasP catP*	
pAP109	P*_xyl_::cas9-opt ΔrasP* P*_gdh_::sgRNA-rasP catP*	
pCE675	P*_xyl_*::*rasP cat*	
pCE620	P*_xyl_*::*cfp-rsiV cat*	
pCE621	P*_xyl_*::c*fp-rsiV*^A69W^ *cat*	
pCE622	P*_xyl_*::*cfp-rsiV*^N71W^ *cat*	
pCE627	P*_xyl_*::*cfp-rsiV*^V67W^ *cat*	

10.1128/msphere.00092-22.3 TABLE S1 Expanded plasmids. Download Table S1, PDF file, 0.2 MB. Copyright © 2022 Pannullo and Ellermeier. 2022 Pannullo and Ellermeier. https://creativecommons.org/licenses/by/4.0/ This content is distributed under the terms of the Creative Commons Attribution 4.0 International license.

10.1128/msphere.00092-22.4 TABLE S2 Oligonucleotides. Download Table S2, PDF file, 0.09 MB. Copyright © 2022 Pannullo and Ellermeier. 2022 Pannullo and Ellermeier. https://creativecommons.org/licenses/by/4.0/ This content is distributed under the terms of the Creative Commons Attribution 4.0 International license.

For xylose-inducible overexpression constructs, genes of interest were amplified using PCR, the oligonucleotides are listed in Table S2. PCR amplicons were then inserted into the plasmid pAP114 at the SacI and BamHI sites, as described previously (47).

### Lysozyme MIC determination.

Overnight cultures of C. difficile were subcultured, grown to late log phase (optical density at 600 nm [OD_600_] of 1.0), and then diluted into TY to 10^6^ CFU/mL. For samples that were preincubated with lysozyme, lysozyme was added at the time and concentration indicated. A series of lysozyme concentrations were prepared in a 96-well plate in 50 μL TY broth. Wells were inoculated with 50 μL of the dilute late-log-phase culture (0.5 × 10^5^ CFU/well) and grown at 37°C for 16 h. Each well was then sampled by removing 10 μL and diluting 1:10 in TY broth, and 5 μL of this dilution was spotted onto TY agar and incubated at 37°C for 24 h. The MIC was defined as the lowest concentration of lysozyme at which 5 or fewer colonies were found per spot.

### Fixation protocol.

Cells were fixed as previously described (48–50). Briefly, a 500 μL aliquot of cells grown in TY broth was added to a 100 μL solution of 16% paraformaldehyde (Alfa Aesar) and 20 μL of 1 M NaPO_4_ buffer (pH 7.4) The sample was mixed, removed from the chamber, and incubated in the dark at room temperature for 60 min. The samples were washed 3 times with phosphate-buffered saline (PBS), resuspended in 100 μL PBS, and left in the dark for a minimum of 3 h to allow for maturation of the chromophore.

### Fluorescence measurements with a plate reader.

Fluorescence from bulk samples was measured using an Infinite M200 Pro plate reader (Tecan) as previously described (49, 50). Briefly, fixed cells in PBS were added to a 96-well microtiter plate (black, flat optical bottom). Fluorescence was recorded as follows: excitation at 554 nm, emission at 610 nm, and gain setting at 140. The cell density (OD_600_) was also recorded and used to normalize the fluorescence reading.

### Immunoblot analysis.

Cultures were grown to mid-log phase (OD_600_ ∼0.7) in TY +1% xylose at which point 20 μg/mL of lysozyme was added to cultures and incubated for 15 min before sample preparation. Samples were suspended in 200 μL of 2× Laemmli sample buffer and sonicated with a Branson Sonifier 450. Samples were electrophoresed on a 15% SDS-polyacrylamide gel which was run at 150V for approximately 90 min. Proteins were then blotted onto a nitrocellulose membrane at 100 mA for 1 h. (Bio-Rad). Nitrocellulose was blocked with 5% bovine serum albumin (BSA) in transfer buffer, and proteins were detected with 1:10,000 anti-GFP antisera. Streptavidin IR680LT (1:10,000) was used to detect the biotinylated protein AccC which served as a loading control and has a size of ∼150 kDa. To detect primary antibodies, the blots were incubated with 1:10,000 goat anti-rabbit IR800CW (Li-Cor) and imaged on an Azure Sapphire imager (Azure Biosystems). All immunoblot assays were performed a minimum of three times with a representative example being shown.
