# Supplementary figures and images for: Activation of the Extracytoplasmic Function σ Factor σV in Clostridioides difficile Requires Regulated Intramembrane Proteolysis of the Anti-σ Factor RsiV
Source: mSphere. 2022 Mar 23;7(2):e00092-22. doi: 10.1128/msphere.00092-22 (PMC9044953; doi:10.1128/msphere.00092-22)

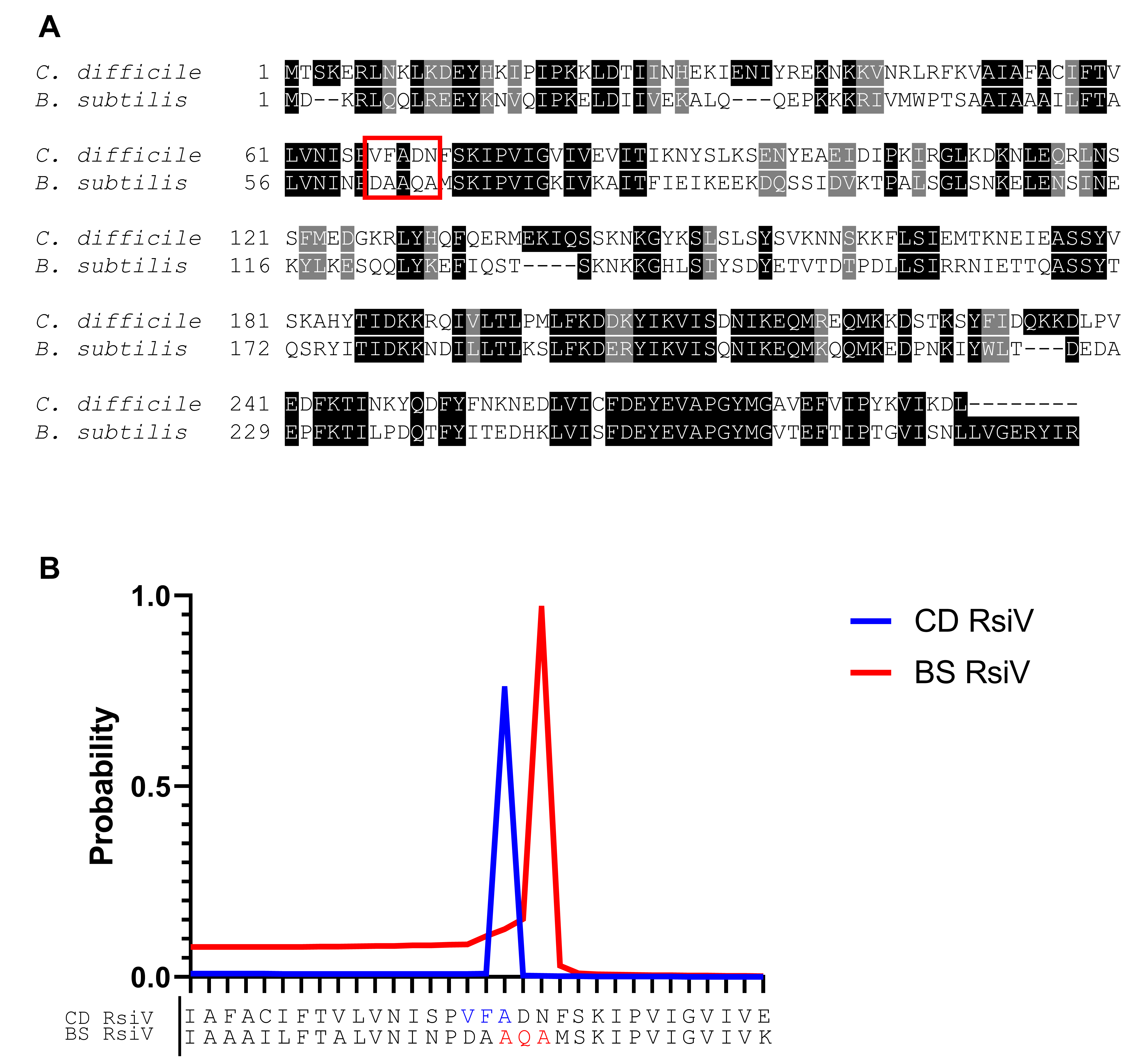

Supplement: FIG S1 [file msphere.00092-22-s0001.tif]

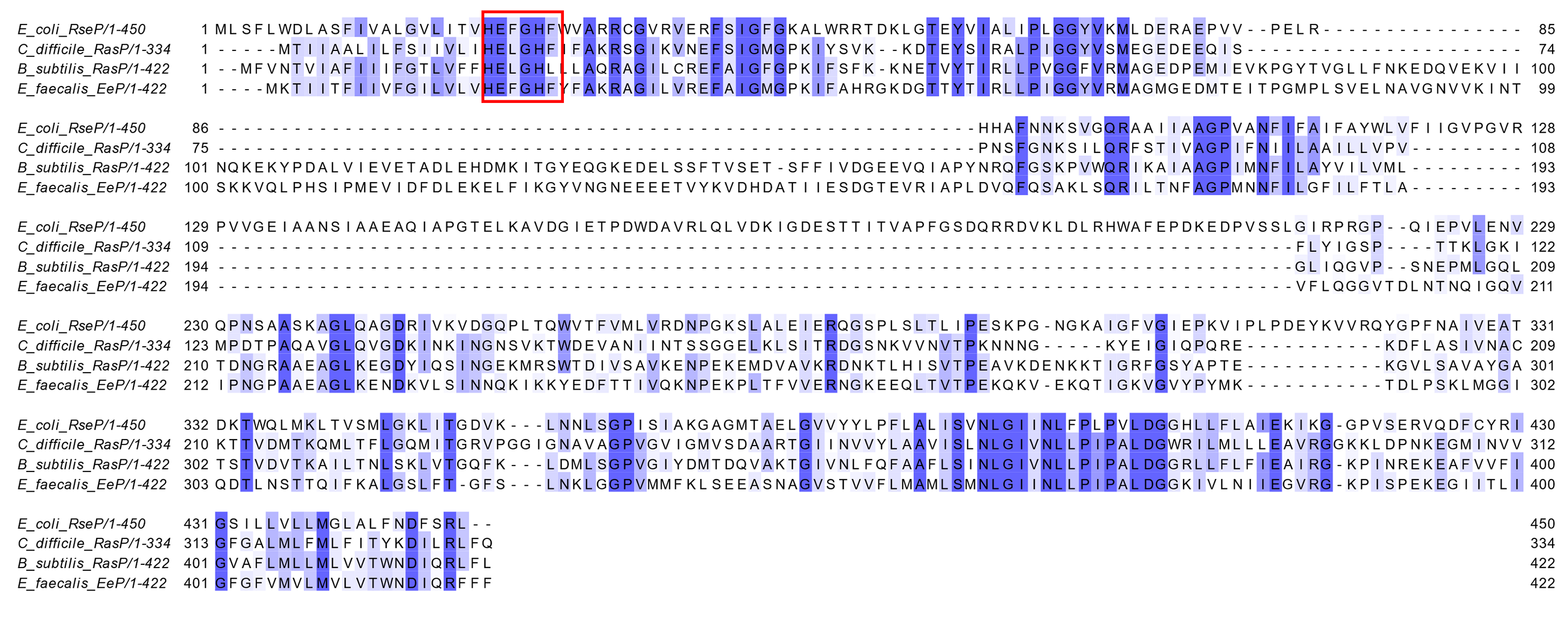

Supplement: FIG S2 [file msphere.00092-22-s0002.tif]
