# Supplementary material for: Activation of the Extracytoplasmic Function σ Factor σV in Clostridioides difficile Requires Regulated Intramembrane Proteolysis of the Anti-σ Factor RsiV
Source: mSphere. 2022 Mar 23;7(2):e00092-22. doi: 10.1128/msphere.00092-22 (PMC9044953; doi:10.1128/msphere.00092-22)
Supplement: TABLE S1 [file msphere.00092-22-s0003.pdf]

TABLE S1. Plasmids used in this study

Table S1. Plasmids

Plasmid	Relevant features	Parent vector	Restriction enzymes to digest parent vector	PCR primers	PCR template	Reference
pRPF185	*E. coli-C. difficile* shuttle vector with tetracycline-inducible promoter; P_{tet} *::gusA cat CD6ori RP4oriT-traJ pMB1 ori*					(54)
pRAN738	P_{pdaV} *::mCherryOpt cat*					(20)
pAP114	P_{xyl} *::mCherryOpt cat*					(47)
pRAN357	P_{tet} *::cfp cat*					(48)
pCE641	P_{xyl} *::cas9-opt ΔrasP* P_{gdh} *::sgRNA-rasP catP*					
pAP109	P_{xyl} *::cas9-opt ΔrasP* P_{gdh} *::sgRNA-rasP catP*	pCE641	KpnI, MluI	sgRNA (4139, 3876)	sgRNA: pIA34	
pCE675	P_{xyl} *::rasP cat*	pAP114	SacI & BamHI	4407-4408	R20291	
pCE620	P_{xyl} *::cfp-rsiV cat*	pAP114	SacI & BamHI	4229-5798 & 5797-4226	CFP: pRAN357 & Rsv: gDNA R20291	
pCE621	P_{xyl} *::cfp-rsiV*^{A69W} *cat*	pAP114	SacI & BamHI	4229-1993 & 1992-4226	pCE620	
pCE622	P_{xyl} *::cfp-rsiV*^{N71W} *cat*	pAP114	SacI & BamHI	4229-1999 & 1998-4226	pCE620	
pCE627	P_{xyl} *::cfp-rsiV*^{V67W} *cat*	pAP114	SacI & BamHI	4229-1974 & 1973-4226	pCE620	
