# Supplementary material for: Activation of the Extracytoplasmic Function σ Factor σV in Clostridioides difficile Requires Regulated Intramembrane Proteolysis of the Anti-σ Factor RsiV
Source: mSphere. 2022 Mar 23;7(2):e00092-22. doi: 10.1128/msphere.00092-22 (PMC9044953; doi:10.1128/msphere.00092-22)
Supplement: TABLE S2 [file msphere.00092-22-s0004.pdf]

Table S2. Oligonucleotides

Oligo	Sequence	Relevant features
4407	cgatagttatgaagtgagcttaaggagggtgaatttatgactataatagccgcatt	Clone RasP into pAP114 digested w/ SacI BamHI
4408	agttttattaaaacttataggatctcattgaaataatcttaatatatctttatatgtgataaaaagca	Clone RasP into pAP114 digested w/ SacI BamHI
4229	cgatagttatgaagtgagctcctgcagtaaaggagaaaattttatg	PCR CFP
5797	atgaattatataagatgaacgcatgcatgacaagcaaagaaaggttaa	PCR CFP
5798	ttaacctttctttgcttgtcatgcatgcgttcatcttatataattcat	PCR RsiV
4226	ttattaaaacttataggatccttacaaatcttttattactttatatggtattacaaactca	PCR RsiV
1992	gtattagtaaatataagtcct tgg tttgcagacaatttttctaag	rsiV V67W
1993	cttagaaaaattgtctgcaaaccaaggacttatatttactaatac	rsiV V67W
1973	aatataagtcctgtattttgggacaatttttctaagatac	rsiV A69W
1974	gtatcttagaaaaattgtcccaaaatacaggacttatatt	rsiV A69W
1998	ataagtcctgtatttgcagactggttttctaagatacctgtaattgg	rsiV N71W
1999	ccaattacaggtatcttagaaaaccagtctgcaaatacaggacttat	rsiV N71W
4127	aaacagctatgaccgcggccgc tctctctctacttccaatattacc	ΔrasP into pCE641 digest with NotI XhoI
4128	aactttcagtttagcggtctgggcgcc tttagtttttagagattttaagaaaatagg	ΔrasP into pCE641 digest with NotI XhoI
4129	ggcgcccagaccgctaaactgaaagtt aaattcaccttcctatttttttatcag	ΔrasP into pCE641 digest with NotI XhoI
4130	cgcatgtctgcaggcctcgag taaaggacttgaagtgatagaagc	ΔrasP into pCE641 digest with NotI XhoI
4139	aattaaactgtaaaggtacc attacaggacgtgtaccagg gttttagagctagaaatagc	sgRNA rasP
